# Enhancing mindfulness in older adulthood: Mixed-method findings from a community-based group intervention

**DOI:** 10.1093/geroni/igaf122.2550

**Published:** 2025-12-31

**Authors:** Tiana Broen, Jonathan Sundby, Christopher Griffith, Leilani Feliciano

**Affiliations:** University of Colorado Colorado Springs, Colorado Springs, Colorado, United States; University of Colorado Colorado Springs, Colorado Springs, Colorado, United States; University of Colorado Colorado Springs, Colorado Springs, Colorado, United States; University of Colorado Colorado Springs Department of Psychology, Colorado Springs, Colorado, United States

## Abstract

Research indicates that mindfulness skills, such as breathing exercises and meditation, effectively reduce psychological symptoms and increase overall well-being. In older adulthood, mindfulness-based interventions are an accessible, cost-effective, and adaptable way to support emotional well-being in everyday life. The current study aimed to examine the efficacy of an 8-week mindfulness skill-building groups on older adults in a community outpatient clinic in the Rocky Mountain Region. Quantitative and qualitative data were gathered across four groups between 2023 and 2024 (N = 22, Mage = 72.13, SD = 5.42, 77.27% female). Participants completed pre- and post-measures of depression, anxiety, stress, well-being, pain, sleep, and trait-mindfulness. To provide a subjective account of specific changes participants had noticed in themselves because of their mindfulness practice, participants were asked to provide narrative feedback, which was recorded and transcribed for thematic analysis. Paired-sample t-tests indicated a significant decrease in perceived stress [t(21) = 3.01, p<.01], depressive symptoms [t(16) = 2.09, p<.05], and anxiety symptoms [t(21) = 3.18, p<.01], as well as an increase in trait mindfulness [t(20) = -4.65, p< .01] and overall well-being [t(21) = -3.24, p<.01]. Thematic analyses were conducted with moderate-good inter-rater reliability (κ=.59-.63), and revealed themes related to the primary outcome variables, in addition to themes of overall effectiveness, interpersonal functioning, group cohesion, mindfulness skills, insight, and positive self-concept. These results indicate that mindfulness-based group interventions can be effective for reducing psychological symptoms, increasing overall well-being, and providing an opportunity for meaningful connection with older adult peers in a group setting.

